# *rac*-11-Selena-12,13-di­aza­bicyclo­[10.3.0]penta­deca-10a(13a),12-dien-1-ol

**DOI:** 10.1107/S2414314621000699

**Published:** 2021-01-26

**Authors:** Heiner Detert, Dieter Schollmeyer

**Affiliations:** a Johannes Gutenberg University Mainz, Department of Chemistry, Duesbergweg 10-14, 55099 Mainz, Germany; Goethe-Universität Frankfurt, Germany

**Keywords:** crystal structure, heterocycles, medium-sized ring, selenium

## Abstract

The title compound, C_12_H_20_N_2_OSe, crystallizes in strands of enanti­omeric mol­ecules connected *via* O—H⋯N hydrogen bonds. There are only slight deviations from an ideal *gauche* conformation in the deca­methyl­ene chain, indicating just a little strain.

## Abstract 2

The title compound crystallizes in strands of enanti­omeric mol­ecules connected *via* O—H⋯N hydrogen bonds. There are only slight deviations from an ideal *gauche* conformation in the deca­methyl­ene chain, indicating just a little strain.

## Graphical abstract

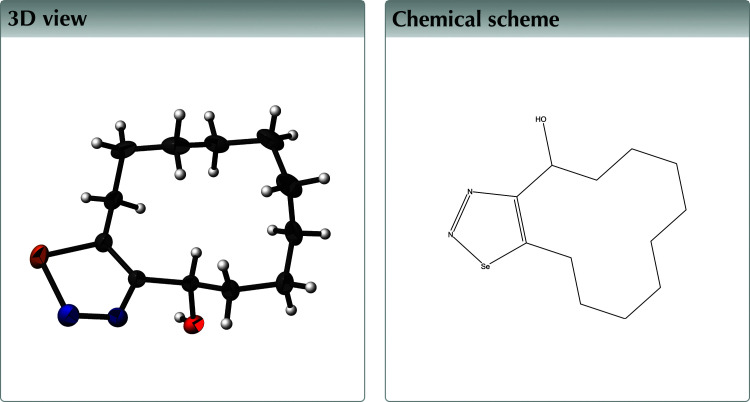

## Structure description

1,2,3-Selena­diazo­les are inter­mediates in the synthesis of other heterocycles (Detert, 2011 ▸), strained (Bissinger *et al.*, 1988 ▸) and functionalized cyclo­alkynes. In addition, they are important for strain-accelerated 3 + 2-cyclo­additions (Ziegler & Wilms, 1950 ▸; Agard *et al.*, 2004 ▸).

**Figure 1:**
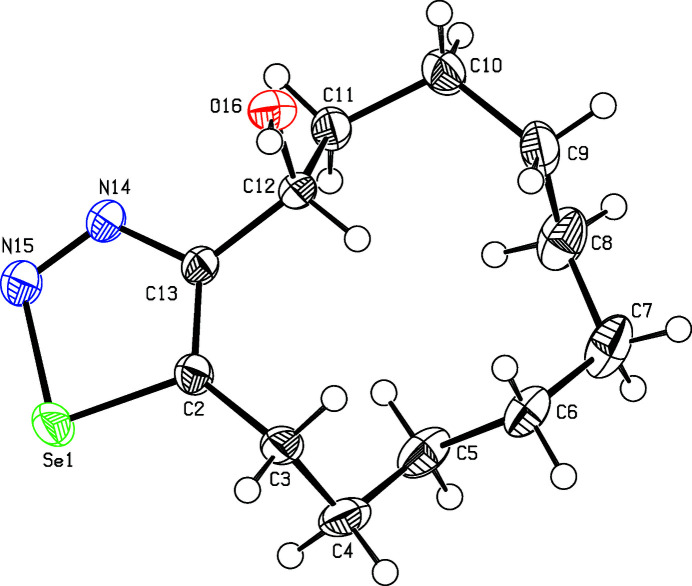
Perspective view of the title compound. Displacement ellipsoids are drawn at the 50% probability level.

**Figure 2:**
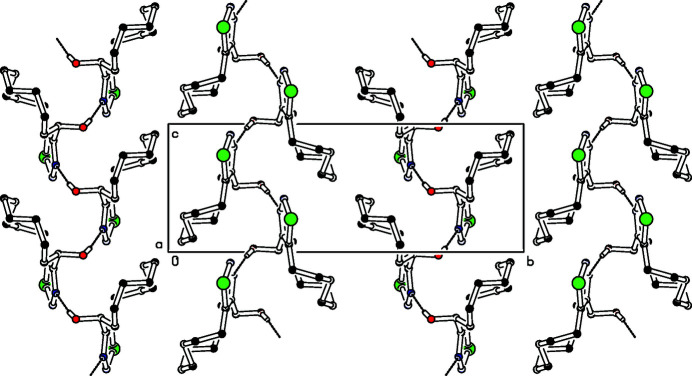
Partial packing diagram of the title compound. View along the *a*-axis. The dotted line indicates the O—H⋯N hydrogen bond.

There is one mol­ecule of the title compound in the asymmetric unit (Fig. 1 ▸), resulting in four mol­ecules filling the unit cell. The crystal is formed from two strands of mol­ecules without directional bonding between the strands (Fig. 2 ▸). Within the strands, the enanti­omeric mol­ecules are connected *via* *c*-glide symmetry. Additionally, the hydrogen bond O16—H16⋯N14 (Table 1 ▸) consolidates the structure. The geometry of the heterocycle matches nearly perfectly that of a recently reported congener (Detert & Schollmeyer, 2020 ▸). Within the deca­methyl­ene tether, strain is only visible at C7—C8—C9—C10: the torsion angle of −149.0 (2)° differs by more than 30° from the ideal *trans* conformation. C—C—C bond angles in the tether are 112–115°, giving further proof of a nearly strain-free ring system.

## Synthesis and crystallization

Acyl­oin condensation of diethyl dodeca­nedioate (Stoll & Rouvé, 1947 ▸; Rühlmann, 1971 ▸), acetyl­ation with acetic anhydride in pyridine, reaction with semicarbazide and oxidation with SeO_2_ (Lalezari *et al.*, 1972 ▸) to acet­oxy­cyclo­dodeceno-1,2,3-selena­diazole followed by amino­lysis of the ester led to the title compound as a viscous oil. Crystallization *via* slow evaporation of a solution in CDCl_3_ gave colorless crystals with m.p. = 380 K. Characterization: ^1^H NMR (CDCl_3_, 400 MHz): 4.98 (*dd*, 1 H), 3.14 (*ddd*, 1 H), 3.01 (*ddd*, 1 H), 2.30–2.00 (*m*, 3 H), 1.88 (*m*, 1 H), 1.62 (*m*, 1 H). 1.55–0.90 (*m*, 13 H). ^13^C-NMR (CDCl_3_, 75 MHz): 163.3 (Se-satellites, *J* = 134 Hz), 162.1 (Se-satellites, *J* = 27 Hz), 37.1, 32.2, 25.0, 24.9, 24.9, 24.3, 24.3, 22.8, 22.8, 22.1. ^77^Se NMR: (CDCl3, 76.4 MHz, Me_2_Se = 0 p.p.m.) δ = 1528.23 p.p.m. MS (EI) *m*/*z* = 174 (9%, M—N_2_—Se), 146 (14%, M—N_2_—Se—C_2_H_4_, 94 (100%).

## Refinement

Crystal data, data collection and structure refinement details are summarized in Table 2 ▸.

## Supplementary Material

Crystal structure: contains datablock(s) I, global. DOI: 10.1107/S2414314621000699/bt4106sup1.cif

Structure factors: contains datablock(s) I. DOI: 10.1107/S2414314621000699/bt4106Isup2.hkl

CCDC reference: 2057509

Additional supporting information:  crystallographic information; 3D view; checkCIF report

## Figures and Tables

**Table 1:** Hydrogen-bond geometry (Å, °)

*D*—H⋯*A*	*D*—H	H⋯*A*	*D*⋯*A*	*D*—H⋯*A*
O16—H16⋯N14^i^	0.77 (3)	2.22 (3)	2.976 (2)	170 (3)

Symmetry code: (i) 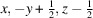 .

**Table 2:** Experimental details

Crystal data	
Chemical formula	C_12_H_20_N_2_OSe
*M*_r_	287.26
Crystal system, space group	Monoclinic, *P*2_1_/*c*
Temperature (K)	193
*a*, *b*, *c* (Å)	8.9261 (5), 19.9277 (9), 7.3829 (4)
β (°)	103.321 (4)
*V* (Å^3^)	1277.91 (12)
*Z*	4
Radiation type	Mo *K*α
μ (mm^−1^)	2.92
Crystal size (mm)	0.71 × 0.26 × 0.20

Data collection	
Diffractometer	Stoe IPDS 2T
Absorption correction	Integration [*X-RED32* (Stoe & Cie, 2019 ▸), absorption correction by Gaussian integration, analogous to Coppens (1970 ▸)]
*T*_min_, *T*_max_	0.294, 0.601
No. of measured, independent and observed [*I* > 2σ(*I*)] reflections	6412, 3020, 2721
*R*_int_	0.017
(sin θ/λ)_max_ (Å^−1^)	0.658

Refinement	
*R*[*F* ^2^ > 2σ(*F* ^2^)], *wR*(*F* ^2^), *S*	0.029, 0.070, 1.14
No. of reflections	3020
No. of parameters	216
H-atom treatment	All H-atom parameters refined
Δρ_max_, Δρ_min_ (e Å^−3^)	0.41, −0.68

Computer programs: *X-AREA* *WinXpose*, *Recipe* and *Integrate* (Stoe & Cie, 2019 ▸), *SIR2004* (Burla *et al.*, 2005 ▸), *SHELXL2018/3* (Sheldrick, 2015 ▸) and *PLATON* (Spek, 2020 ▸).

## full crystallographic data

### Crystal data

**Table:**

C_12_H_20_N_2_OSe	*F*(000) = 592
*M**_r_* = 287.26	*D*_x_ = 1.493 Mg m^−^^3^
Monoclinic, *P*2_1_/*c*	Mo *K*α radiation, λ = 0.71073 Å
*a* = 8.9261 (5) Å	Cell parameters from 9207 reflections
*b* = 19.9277 (9) Å	θ = 2.6–28.4°
*c* = 7.3829 (4) Å	µ = 2.92 mm^−^^1^
β = 103.321 (4)°	*T* = 193 K
*V* = 1277.91 (12) Å^3^	Block, colourless
*Z* = 4	0.71 × 0.26 × 0.20 mm

### Data collection

**Table:**

Stoe IPDS 2T diffractometer	3020 independent reflections
Radiation source: sealed X-ray tube, 12 x 0.4 mm long-fine focus	2721 reflections with *I* > 2σ(*I*)
Detector resolution: 6.67 pixels mm^-1^	*R*_int_ = 0.017
rotation method, ω scans	θ_max_ = 27.9°, θ_min_ = 2.6°
Absorption correction: integration [X-Red32 (Stoe & Cie, 2019), absorption correction by Gaussian integration, analogous to Coppens (1970)]	*h* = −11→10
*T*_min_ = 0.294, *T*_max_ = 0.601	*k* = −24→26
6412 measured reflections	*l* = −9→9

### Refinement

**Table:**

Refinement on *F*^2^	Primary atom site location: structure-invariant direct methods
Least-squares matrix: full	Hydrogen site location: difference Fourier map
*R*[*F*^2^ > 2σ(*F*^2^)] = 0.029	All H-atom parameters refined
*wR*(*F*^2^) = 0.070	*w* = 1/[σ^2^(*F*_o_^2^) + (0.0304*P*)^2^ + 0.7552*P*] where *P* = (*F*_o_^2^ + 2*F*_c_^2^)/3
*S* = 1.14	(Δ/σ)_max_ = 0.001
3020 reflections	Δρ_max_ = 0.41 e Å^−^^3^
216 parameters	Δρ_min_ = −0.67 e Å^−^^3^
0 restraints	

### Special details

**Table:**

Geometry. All esds (except the esd in the dihedral angle between two l.s. planes) are estimated using the full covariance matrix. The cell esds are taken into account individually in the estimation of esds in distances, angles and torsion angles; correlations between esds in cell parameters are only used when they are defined by crystal symmetry. An approximate (isotropic) treatment of cell esds is used for estimating esds involving l.s. planes.
Refinement. Hydrogen atoms were refined with isotropic displacement parameters constraining the *U*s of two H atoms bonded to the same C atom to the same value.

### Fractional atomic coordinates and isotropic or equivalent isotropic displacement parameters (Å^2^)

**Table:**

	*x*	*y*	*z*	*U*_iso_*/*U*_eq_	
Se1	0.82417 (2)	0.15493 (2)	0.75710 (3)	0.03612 (8)	
C2	0.6550 (2)	0.16090 (9)	0.5593 (2)	0.0263 (3)	
C3	0.6625 (2)	0.15035 (10)	0.3601 (3)	0.0296 (4)	
H3A	0.755 (3)	0.1703 (11)	0.344 (3)	0.034 (4)*	
H3B	0.579 (3)	0.1750 (12)	0.279 (3)	0.034 (4)*	
C4	0.6544 (3)	0.07597 (12)	0.3019 (3)	0.0389 (5)	
H4A	0.687 (3)	0.0747 (13)	0.183 (4)	0.044 (5)*	
H4B	0.730 (3)	0.0518 (13)	0.394 (4)	0.044 (5)*	
C5	0.4957 (3)	0.04424 (11)	0.2800 (3)	0.0378 (5)	
H5A	0.462 (3)	0.0536 (13)	0.390 (4)	0.046 (5)*	
H5B	0.499 (3)	−0.0049 (14)	0.271 (4)	0.046 (5)*	
C6	0.3781 (3)	0.06857 (11)	0.1084 (3)	0.0348 (4)	
H6A	0.372 (3)	0.1168 (12)	0.112 (3)	0.034 (4)*	
H6B	0.420 (3)	0.0588 (11)	0.000 (3)	0.034 (4)*	
C7	0.2171 (3)	0.03777 (13)	0.0816 (3)	0.0470 (6)	
H7A	0.221 (3)	−0.0077 (15)	0.071 (4)	0.058 (6)*	
H7B	0.149 (3)	0.0529 (14)	−0.043 (4)	0.058 (6)*	
C8	0.1357 (3)	0.05394 (12)	0.2377 (3)	0.0459 (5)	
H8A	0.200 (3)	0.0389 (14)	0.359 (4)	0.056 (6)*	
H8B	0.040 (3)	0.0265 (14)	0.224 (4)	0.056 (6)*	
C9	0.0886 (2)	0.12727 (13)	0.2466 (3)	0.0388 (5)	
H9A	0.151 (3)	0.1562 (12)	0.186 (4)	0.041 (5)*	
H9B	−0.010 (3)	0.1333 (13)	0.172 (4)	0.041 (5)*	
C10	0.0892 (2)	0.15211 (12)	0.4425 (3)	0.0356 (4)	
H10A	0.049 (3)	0.1978 (14)	0.435 (3)	0.042 (5)*	
H10B	0.016 (3)	0.1240 (13)	0.494 (3)	0.042 (5)*	
C11	0.2460 (2)	0.15032 (10)	0.5800 (3)	0.0286 (4)	
H11A	0.282 (3)	0.1034 (13)	0.606 (3)	0.036 (4)*	
H11B	0.238 (3)	0.1680 (12)	0.694 (4)	0.036 (4)*	
C12	0.36991 (19)	0.19034 (9)	0.5151 (2)	0.0235 (3)	
H12	0.372 (2)	0.1780 (10)	0.388 (3)	0.019 (5)*	
C13	0.52951 (19)	0.17624 (9)	0.6290 (2)	0.0229 (3)	
N14	0.55574 (18)	0.18287 (8)	0.8195 (2)	0.0273 (3)	
N15	0.6932 (2)	0.17459 (9)	0.9149 (2)	0.0337 (4)	
O16	0.33526 (16)	0.26009 (7)	0.52548 (19)	0.0298 (3)	
H16	0.385 (3)	0.2790 (13)	0.471 (4)	0.042 (7)*	

### Atomic displacement parameters (Å^2^)

**Table:**

	*U*^11^	*U*^22^	*U*^33^	*U*^12^	*U*^13^	*U*^23^
Se1	0.02420 (11)	0.05973 (15)	0.02309 (11)	0.00772 (8)	0.00266 (7)	0.00148 (8)
C2	0.0242 (8)	0.0346 (9)	0.0195 (8)	0.0024 (7)	0.0041 (6)	0.0016 (6)
C3	0.0259 (9)	0.0447 (11)	0.0199 (8)	0.0060 (8)	0.0087 (7)	0.0013 (7)
C4	0.0465 (12)	0.0474 (12)	0.0247 (9)	0.0209 (10)	0.0122 (8)	−0.0004 (8)
C5	0.0617 (14)	0.0285 (10)	0.0255 (9)	0.0074 (9)	0.0147 (9)	0.0011 (7)
C6	0.0507 (12)	0.0313 (10)	0.0237 (9)	−0.0019 (8)	0.0116 (8)	−0.0038 (7)
C7	0.0634 (15)	0.0433 (13)	0.0350 (11)	−0.0173 (11)	0.0127 (10)	−0.0138 (10)
C8	0.0552 (13)	0.0454 (13)	0.0395 (12)	−0.0232 (11)	0.0162 (10)	−0.0096 (9)
C9	0.0289 (10)	0.0558 (13)	0.0286 (9)	−0.0081 (9)	0.0002 (8)	−0.0025 (9)
C10	0.0237 (9)	0.0506 (12)	0.0325 (10)	−0.0025 (8)	0.0063 (7)	−0.0029 (8)
C11	0.0244 (8)	0.0383 (10)	0.0235 (8)	−0.0032 (7)	0.0061 (7)	−0.0013 (7)
C12	0.0227 (8)	0.0293 (9)	0.0191 (7)	0.0020 (6)	0.0060 (6)	−0.0017 (6)
C13	0.0239 (8)	0.0269 (8)	0.0183 (7)	−0.0003 (6)	0.0058 (6)	−0.0006 (6)
N14	0.0288 (7)	0.0346 (8)	0.0187 (7)	0.0013 (6)	0.0059 (6)	−0.0008 (6)
N15	0.0319 (8)	0.0479 (10)	0.0204 (7)	0.0047 (7)	0.0042 (6)	0.0000 (6)
O16	0.0314 (7)	0.0297 (7)	0.0303 (7)	0.0044 (5)	0.0113 (5)	0.0029 (5)

### Geometric parameters (Å, º)

**Table:**

Se1—C2	1.8467 (18)	C8—C9	1.526 (4)
Se1—N15	1.8723 (17)	C8—H8A	0.99 (3)
C2—C13	1.371 (2)	C8—H8B	1.00 (3)
C2—C3	1.502 (2)	C9—C10	1.528 (3)
C3—C4	1.540 (3)	C9—H9A	0.98 (3)
C3—H3A	0.95 (2)	C9—H9B	0.93 (3)
C3—H3B	0.97 (2)	C10—C11	1.529 (3)
C4—C5	1.525 (3)	C10—H10A	0.98 (3)
C4—H4A	0.99 (3)	C10—H10B	1.00 (3)
C4—H4B	0.97 (3)	C11—C12	1.527 (2)
C5—C6	1.526 (3)	C11—H11A	0.99 (2)
C5—H5A	0.94 (3)	C11—H11B	0.93 (3)
C5—H5B	0.98 (3)	C12—O16	1.430 (2)
C6—C7	1.533 (3)	C12—C13	1.505 (2)
C6—H6A	0.96 (2)	C12—H12	0.97 (2)
C6—H6B	0.97 (2)	C13—N14	1.378 (2)
C7—C8	1.532 (3)	N14—N15	1.276 (2)
C7—H7A	0.91 (3)	O16—H16	0.77 (3)
C7—H7B	1.03 (3)		
			
C2—Se1—N15	87.97 (7)	C9—C8—H8A	111.0 (17)
C13—C2—C3	128.55 (16)	C7—C8—H8A	109.7 (17)
C13—C2—Se1	107.90 (12)	C9—C8—H8B	106.9 (17)
C3—C2—Se1	123.55 (13)	C7—C8—H8B	110.7 (17)
C2—C3—C4	113.45 (16)	H8A—C8—H8B	103 (2)
C2—C3—H3A	107.5 (14)	C8—C9—C10	114.19 (19)
C4—C3—H3A	110.8 (14)	C8—C9—H9A	110.9 (14)
C2—C3—H3B	109.0 (14)	C10—C9—H9A	111.2 (16)
C4—C3—H3B	109.5 (14)	C8—C9—H9B	109.3 (16)
H3A—C3—H3B	106 (2)	C10—C9—H9B	108.2 (16)
C5—C4—C3	114.30 (16)	H9A—C9—H9B	102 (2)
C5—C4—H4A	110.8 (15)	C9—C10—C11	115.09 (17)
C3—C4—H4A	105.5 (15)	C9—C10—H10A	109.0 (15)
C5—C4—H4B	110.2 (15)	C11—C10—H10A	109.2 (15)
C3—C4—H4B	107.5 (15)	C9—C10—H10B	108.6 (15)
H4A—C4—H4B	108 (2)	C11—C10—H10B	108.1 (14)
C4—C5—C6	113.62 (17)	H10A—C10—H10B	107 (2)
C4—C5—H5A	107.3 (16)	C12—C11—C10	113.45 (16)
C6—C5—H5A	110.8 (16)	C12—C11—H11A	109.0 (14)
C4—C5—H5B	112.2 (16)	C10—C11—H11A	111.0 (14)
C6—C5—H5B	106.6 (15)	C12—C11—H11B	107.5 (15)
H5A—C5—H5B	106 (2)	C10—C11—H11B	109.8 (15)
C5—C6—C7	115.13 (19)	H11A—C11—H11B	106 (2)
C5—C6—H6A	108.9 (14)	O16—C12—C13	109.89 (14)
C7—C6—H6A	110.5 (14)	O16—C12—C11	108.02 (14)
C5—C6—H6B	107.3 (14)	C13—C12—C11	112.81 (14)
C7—C6—H6B	109.7 (14)	O16—C12—H12	110.3 (12)
H6A—C6—H6B	104.7 (19)	C13—C12—H12	105.6 (12)
C8—C7—C6	114.35 (18)	C11—C12—H12	110.2 (12)
C8—C7—H7A	107.9 (19)	C2—C13—N14	116.31 (15)
C6—C7—H7A	110.7 (19)	C2—C13—C12	125.62 (15)
C8—C7—H7B	109.3 (17)	N14—C13—C12	117.96 (14)
C6—C7—H7B	109.9 (16)	N15—N14—C13	117.79 (15)
H7A—C7—H7B	104 (2)	N14—N15—Se1	110.03 (12)
C9—C8—C7	114.6 (2)	C12—O16—H16	107 (2)
			
N15—Se1—C2—C13	0.38 (14)	C10—C11—C12—C13	−167.50 (16)
N15—Se1—C2—C3	−179.79 (17)	C3—C2—C13—N14	179.73 (18)
C13—C2—C3—C4	−96.5 (2)	Se1—C2—C13—N14	−0.5 (2)
Se1—C2—C3—C4	83.7 (2)	C3—C2—C13—C12	−4.2 (3)
C2—C3—C4—C5	72.5 (2)	Se1—C2—C13—C12	175.63 (14)
C3—C4—C5—C6	71.9 (2)	O16—C12—C13—C2	−108.7 (2)
C4—C5—C6—C7	−179.99 (18)	C11—C12—C13—C2	130.74 (19)
C5—C6—C7—C8	62.7 (3)	O16—C12—C13—N14	67.4 (2)
C6—C7—C8—C9	68.6 (3)	C11—C12—C13—N14	−53.2 (2)
C7—C8—C9—C10	−149.0 (2)	C2—C13—N14—N15	0.3 (3)
C8—C9—C10—C11	62.1 (3)	C12—C13—N14—N15	−176.12 (17)
C9—C10—C11—C12	57.0 (2)	C13—N14—N15—Se1	0.1 (2)
C10—C11—C12—O16	70.83 (19)	C2—Se1—N15—N14	−0.26 (15)

### Hydrogen-bond geometry (Å, º)

**Table:**

*D*—H···*A*	*D*—H	H···*A*	*D*···*A*	*D*—H···*A*
O16—H16···N14^i^	0.77 (3)	2.22 (3)	2.976 (2)	170 (3)

Symmetry code: (i) *x*, −*y*+1/2, *z*−1/2.
